# Supplementary material for: The ectoparasitic seal louse, Echinophthirius horridus, relies on a sealed tracheal system and spiracle closing apparatus for underwater respiration
Source: Commun Biol. 2025 Jun 3;8:852. doi: 10.1038/s42003-025-08285-4 (PMC12134155; doi:10.1038/s42003-025-08285-4)
Supplement: Supplementary file 1 — Supplementary Information [file 42003_2025_8285_MOESM1_ESM.pdf]

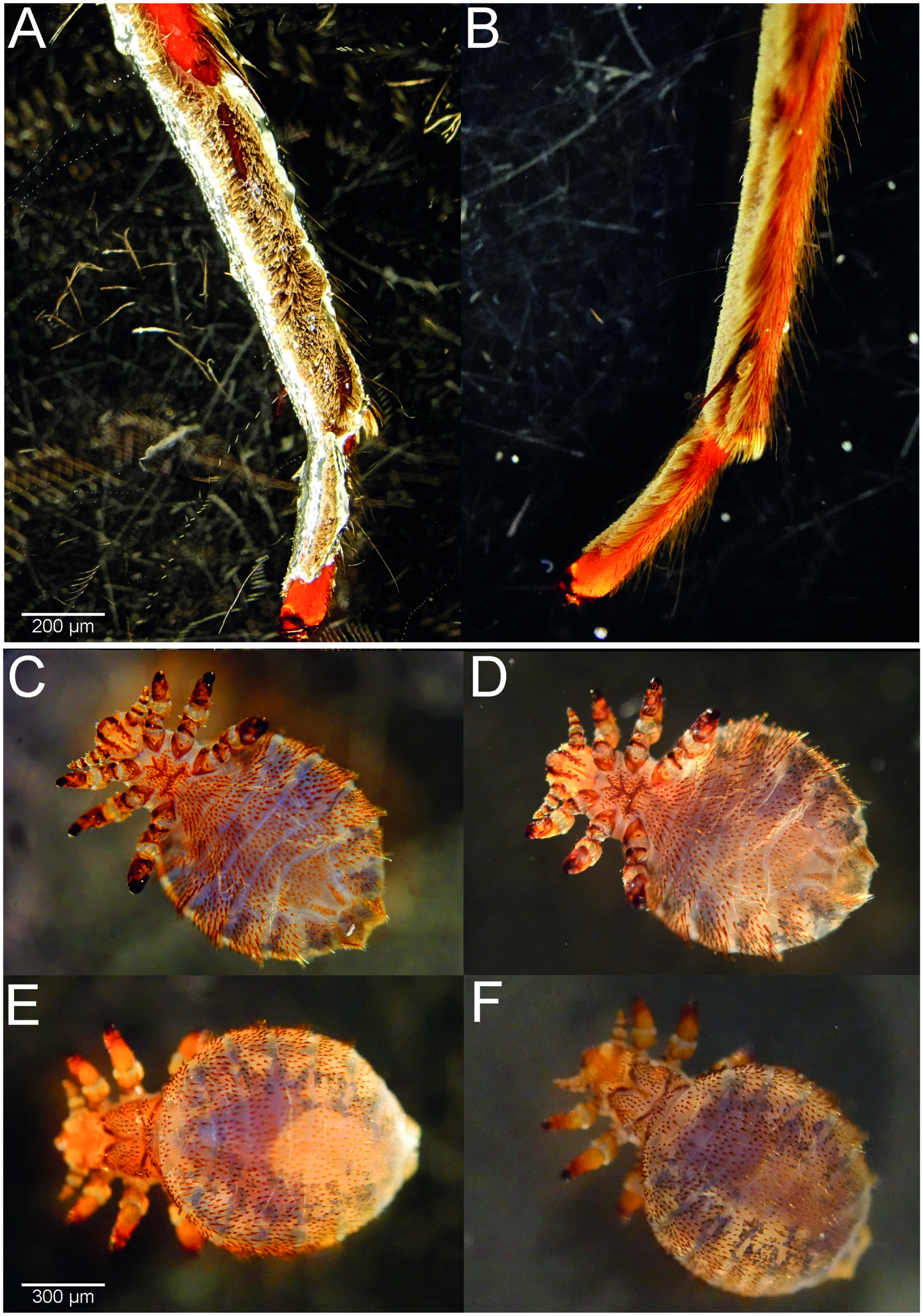

Supplementary Figure 1: Microscopic examination of plastrons in *Cupiennius salei* (A-B) and *E. horridus* (C-F). A) Middle leg of *C. salei* in Baltic Sea water with visible plastron (shiny surface). B) Middle leg of *C. salei* in mixture of Baltic Sea water and 0.1% Triton X with dissolved plastron. C, E) *Echinophthirius horridus* from ventral and dorsal view in Baltic Sea water with no visible plastron. D, F) *Echinophthirius horridus* from ventral and dorsal view in mixture of Baltic Sea water and 0.1% Triton X with no visible plastron. The seal louse was observed in a living state.
