# Supplementary material for: The ectoparasitic seal louse, Echinophthirius horridus, relies on a sealed tracheal system and spiracle closing apparatus for underwater respiration
Source: Commun Biol. 2025 Jun 3;8:852. doi: 10.1038/s42003-025-08285-4 (PMC12134155; doi:10.1038/s42003-025-08285-4)
Supplement: Supplementary file 2 — Description of Additional Supplementary Files [file 42003_2025_8285_MOESM2_ESM.docx]

Description of Additional Supplementary Files

**File Name:** Supplementary Data 1

**Description:** Dataset of the measured and calculated respiratory volume, masses, calibrations, and experimental settings.

**File Name:** Supplementary Data 2

**Description:** Dataset of the measured diameters of seal louse and head louse trachea of different orders (1st, 2nd, 3rd order trachea and trachea in the head region).

**File Name:** Supplementary Data 3

**Description:** Table with all publications and data used for the creation of Figure 7 based on literature for respiration in insects.

**File Name:** Supplementary Code

**Description:** R scripts used for statistical tests and graphs.
