# Supplementary material for: The ectoparasitic seal louse, Echinophthirius horridus, relies on a sealed tracheal system and spiracle closing apparatus for underwater respiration
Source: Commun Biol. 2025 Jun 3;8:852. doi: 10.1038/s42003-025-08285-4 (PMC12134155; doi:10.1038/s42003-025-08285-4)
Supplement: Supplementary file 6 — Supplementary Code [file 42003_2025_8285_MOESM6_ESM.pdf]

```
##################################################################################
##################################################################################
############
```

This R-script is part of the manuscript "The ectoparasitic seal louse, *Echinophthirius horridus,* relies on a sealed tracheal system and spiracle closing apparatus for underwater respiration"

```
+++++++++ ANOVA Buoyancy
+++++++++++++++++++++++++++++++++++++++++++++++++++++++++++++++++++++++++++++++++++++
++++++++++++++++++++++++++++++++++++++++++++

##################################################################################
##################################################################################
############

# Clear workspace

rm(list = ls())

library(ggplot2)

library(tidyverse)

library(ggpubr)

library(rstatix)

# Import dataset

library(readxl)

Supplementary_Data1 <- read_excel("F:/Manuskripte/Buoyancy/Datens?tze/
Supplementary_Data1",

            sheet = "ANOVA")

View(Supplementary_Data1)

attach(Supplementary_Data1)
```

```
# Normality assumption

shapiro.test(S3_Buoyancy$Respiration)


# Data preparation
Supplementary_Data1 <- Supplementary_Data1 %>%
  gather(key = "Treatment", value = "Respiration", Natural, Triton, Washed) %>%
  convert_as_factor(Species_shortcut, Iteration)
data.frame(head(Supplementary_Data1))


# Summary statistics
Supplementary_Data1 %>%
  group_by(Treatment) %>%
  get_summary_stats(Respiration, type = "mean_sd")


# Check for outliers
Supplementary_Data1 %>%
  group_by(Treatment) %>%
  identify_outliers(Respiration)

# Normality assumption
Supplementary_Data1 %>%
  group_by(Treatment) %>%
  shapiro_test(Respiration) # normally distributed -> One-way ANOVA


# One-way ANOVA
one.way <- aov(Respiration ~ Treatment, data = Supplementary_Data1)
```

```
summary(one.way)

##################################################################################
##################################################################################
############
```

This R-script is part of the manuscript "The ectoparasitic seal louse, *Echinophthirius horridus,* relies on a sealed tracheal system and spiracle closing apparatus for underwater respiration"

```
+++++++++ Boxplot Buoyancy
++++++++++++++++++++++++++++++++++++++++++++++++++++++++++++++++++++++++++++++++++
++++++++++++++++++++++++++++++++++++++++++

##################################################################################
##################################################################################
############

# Clear workspace

rm(list = ls())

library(ggplot2)

# Import dataset

library(readxl)

Supplementary_Data1 <- read_excel("F:/Manuskripte/Buoyancy/Datens?tze/
Supplementary_Data1.xlsx",

            sheet= "Calculations")

View(Supplementary_Data1)
```

```
attach(Supplementary_Data1)

# Make boxplot

pd = position_dodge(width = 1.1)

p <- ggplot(Supplementary_Data1, aes(x=Iteration_of_measurement, y=Vresp_in_mm3_calibrated))
+
  stat_boxplot(geom='errorbar', position = pd, width=0.1) +
  geom_boxplot(width = 0.7, position=position_dodge(width = 1.1)) +
  stat_summary(fun = mean, geom = "point", color = "firebrick", shape = 17, size = 2, position =
position_dodge(width = 1.1)) +
  scale_fill_manual(values=c("#999999", "#E69F00")) +
  theme_classic() +
  labs(y = "Vresp in mm3") +
  theme(plot.title = element_text(hjust = 0.5, size = 16, face = "bold"), plot.caption =
element_text(hjust = 0, size = 10), plot.tag = element_text(size = 16, face = "bold")) + # adjust title
and caption position
  scale_y_continuous(expand = c(0, 0), limits = c(0, 0.1))
p
```

```
##################################################################################
##################################################################################
############
```

This R-script is part of the manuscript "The ectoparasitic seal louse, *Echinophthirius horridus,* relies on a sealed tracheal system and spiracle closing apparatus for underwater respiration"

```
+++++++++ Lineplot Buoyancy
+++++++++++++++++++++++++++++++++++++++++++++++++++++++++++++++++++++++++++++++++
+++++++++++++++++++++++++++++++++++++++++

##################################################################################
##################################################################################
############

# Clear workspace

rm(list = ls())



library(ggplot2)



# Import dataset


library(readxl)

Supplementary_Data1 <- read_excel("F:/Manuskripte/Buoyancy/Datens?tze/
Supplementary_Data1",

                      sheet = "Progression_curve")

View(Supplementary_Data1)



attach(Supplementary_Data1)
```

```
# Make plot


p <- ggplot(Supplementary_Data1, aes(x = time, y = Vresp_in_mm3_calibrated, colour=Iteration)) +
  geom_point(size=2) +
  stat_smooth(se=F) +
  theme_classic()



p
```

###
###
############

This R-script is part of the manuscript "The ectoparasitic seal louse, *Echinophthirius horridus,* relies on a sealed tracheal system and spiracle closing apparatus for underwater respiration"

+++++++++ Scatterplot comparison of tracheal volume
+++
+++

###
###
############

```
# Clear workspace

rm(list = ls())


library(ggplot2)
```

```
# Import dataset

library(readxl)

Supplementary_Data3 <- read_excel("F:/Manuskripte/Buoyancy/ Supplementary_Data3")

View(Supplementary_Data3)



attach(Supplementary_Data3)



# Make barplot


tracheal.data <- data.frame(

  tracheal_ID <- c(1:10),

  Species_Name <- c("Tribolium_castaneum","Pediculus_humanus_capitis","Echinophthirius_horridus","Tenebrio_molitor","Calliphora_vicina","Carausius_morosus","Schistocerca_americana","Blaptica_duplica","Eleodes_obscura","Manduca_sexta"),

  Volume <- c(0.5, 0.27, 0.22, 2.76, 49.15, 1.3, 40, 12, 4.8, 7.5))


# Plot the bar chart

barplot(Volume,names.arg=Species_Name,xlab="Species",

       ylab="Tracheal volume",col="black")
```
